# Nature and Treatment of Decay of the Teeth

**Published:** 1867-07

**Authors:** H. R. Noel


### ARTICLE III.

### *Nature and Treatment of Decay of the Teeth.*

A Review of Dr. Arthur's Monograph on Decay of the Teeth. By Professor H. R. Noel.

We have read, and propose to review this work, as it is eminently practical and addressed to the public, as well as to the profession. The subject is one of the utmost moment, and the earnestness of firmly settled conviction marks the entire article. The author does not claim originality, but the practical importance is urged, and he brings to bear upon the question the results of 26 years of careful study and thoughtful experience.

The one idea, upon which the monograph is based, the one idea importunately urged upon the public and the profession is the

THE PROPHYLACTIC TREATMENT OF DECAY.

These are not the words of the author, but they embody his idea.

From the very nature of ordinary treatment, when necessarily resorted to in common practice; the fatigue, annoyance, pain, &c., under the hands of the operator; the reminiscences of shooting pains and swollen faces; the agony of sleepless nights, we instinctively turn to the study of Prophylaxis, as agreeable and cheering at least to the patient, if not to the practitioner. Filing, separating, &c., are carefully considered, and their value placed far higher than is generally acknowledged.

The views, ideas, experiences, &c., of the author can be partially expressed by a series of propositions, embracing the leading points.

I.—"If decay, existing upon teeth in contact be removed by file or otherwise, and the teeth permanently separated, the decay will not return unless in cases of extreme predisposition to decay, or extreme negligence in care of the teeth."

II.—"That if decay occur on the surfaces in contact of the incisor teeth of a child before it is twelve years old, all the teeth, with the exception in most cases of the incisor teeth of the lower jaw, will decay sooner or later at the same places, (i e. "points of contact.") This rule may not be absolutely invariable, but the exceptions are rare."—(Page 48.)

III.—"That this is the whole explanation of decay of teeth: they are made up of a substance capable of being decomposed by acids, which either exist or are formed in the mouth, but so feeble in power as to require to be retained for some time in contact with them to produce the effect which is called decay."—(Page 13.)

IV.—"At and near the points where the contiguous teeth touch each other, the secretions of the mouth are, at all times held in contact with the enamel. Particles of food are frequently forced between them, and remain until they are entirely decomposed. In this manner, such agents capable of destroying the enamel, as may be present in the mouth, are held in contact with the part of the tooth designated, as effectually as if the enamel were defective."

V.—"In order to prevent the entire loss of the teeth, the decomposed parts must be removed, and the affected surfaces left in such a condition as not to afford lodgement to the food, or to the secretions of the mouth."—(Page 26.)

VI.—"There is but one way in which this can be accomplished; the surfaces of the teeth on which the decomposed spots appear, must be cut away so that they cannot again come in contact."—(Page 26.)

VII.—"After cutting, filing, &c., a change takes place in the structure of the dentine, when left exposed, which in time renders it quite as capable of resisting attacks of decay as the enamel itself."—(Page 33.)

VIII.—"But the most important reason that can be urged in favor of the treatment proposed is, that by its means the teeth are more certainly preserved, than by filling them with any substance now known."—(Page 37.)

IX.—"An inevitable deduction of the author's from the above propositions is "that in all cases coming under proposition No. II., the separation of the teeth before puberty at least, is the true method of treatment; and saves pain, annoyance and expense while the teeth are preserved."

Such are the leading points advanced; these will be developed and others introduced as we proceed. The style of the work is plain, clear, inviting; no ambiguity of either ideas or expressions; no attempt at a learned disquisition, but a practical essay intended for the popular mind and so adapted. We propose first to examine filing, cutting, &c., in the light of some of our best authorities, and then review the work more closely.

As is evident, the author is a strong advocate of this measure, let us see what writers support his view.

Dr. H. H. Hayden practiced this over half a century ago, and in many instances these patients have passed recently under the author's notice, and he has carefully examined them; many of them now living in Baltimore, still retain traces of the file, and the work has faithfully stood the test of time. Prof. C. A. Harris in his Principles and Practice of Dental Surgery, most cordially endorses a paper by his brother, Dr. Jno. Harris; the paper was published in the September No. of Vol. V. of the American Journal of Dental Science, and from which we make quotations.

"That an experience obtained from 23 years of constant practice has fully convinced me, not only of the propriety but of the absolute necessity in the treatment of caries of the lateral surfaces of the teeth, of employing the file."

Again, and in forcible language, Dr. Jno. Harris gives us the following

"The principal, and I believe only objection, urged against filing teeth is based upon the *erroneous belief*, that the loss of any part of the enamel of these organs must necessarily result in their destruction. Why do the negroes of Abysinnia and Brahmins of India have such fine teeth, since it is well known they file their front teeth to sharp points, removing of course both enamel and dentine to some considerable extent? Yet travellers often speak of their fine teeth."

Mr. Jno. Tomes in his Dental Surgery, also approves of filing, cutting, &c., though he cautions against leaving a rough surface. Upon page 381, he makes the following statement:

In treatment of simple caries two methods are employed. The removal of the diseased, together with the surrounding healthy tissue, so such an extent as to leave a perfectly smooth surface constitutes one method."

It is therefore one of the acknowledged methods of treating caries Again page 383:

"Nature sometimes performs for herself an operation analogous to filing, when properly performed, both as regards its physical peculiarity and its results. The walls of a broad but shallow cavity produced by caries break down, the softened tissues are exposed to friction, and rubbed away, till at last the hard dentine is reached; this *becomes highly polished and endures for an indefinite time unaltered.*"

The filing therefore is endorsed. He mentions the same change in the Dentine, that our author also mentions; a condensing and hardening.

We have, therefore, Drs. Hayden, J. and C. A. Harris and Mr. Tomes in favor of this treatment. The question then is, not its use but the extent of its use; not is this a valuable and an allowable method, but to what extent can this be practiced? Have the members of the profession, as yet, fully appreciated the true value of this practice.

By filing, we do not intend simply the use of the file only—but include all instruments used for the purpose of removing superficial caries from, and separation of, teeth in approximation. The term filing, is therefore more of a representative word, adopted for convenience. A discussion might arise here, as to the theoretical and practical value of enamel, the two not being identical by any means; this we pass over for the present, as we shall other questions such as—What is the essential nature of caries? Does it always commence upon the exterior? Is caries ever found in the internal structure of a tooth, without an opening leading to the interior from the exterior, and by which said opening the fluids of the mouth gain access to the cavity, and by which said fluids this cavity was itself formed? Can caries be produced by interstitial death of either dentine or enamel? Do enamel and dentine possess enough of vitality, normally, to warrant us in applying the phrase interstitial death to them? Are dentine and enamel truly vitalized structures? Do they undergo waste and repair? Are they liable to any changes after being once developed, and if so—to what extent are they liable and what is the nature of the changes? These are questions yet to be solved, and a future article will be devoted to them. Their discussion in full, would be out of place in the hurried review we are making, and only when they bear upon the subject directly in hand, shall we again notice them, and then in as concise a manner as possible. The lengthy discussion must be reserved for a future article, when we shall endeavor to develope the physiological phase of the subject.

We proceed to a more thorough examination of the monograph.

The first chapter, embracing some eight or nine pages, contains the generally received opinions upon the anatomy of the teeth. The author is concise and clear, and the whole chapter given in a form likely to be popular with the general reader. Upon this we make no comments, as none are required at present.

Chapter II. is upon "Decay of Teeth," embracing also some eight or nine pages, and may be summed up in a condensed form as follows:

The cause of decay is an acid acting slowly but continuously upon the enamel. The mucous secretion of the mouth is acid, the saliva is alkaline, therefore neutralizing to a certain extent; but defective enamel and contiguous teeth may retain the acid mucus long enough to corrode and destroy the organs. Particles of food lodging between the teeth and decomposing, produce the same result, hence the frequency of caries at these points. Defective enamel and contiguity of teeth are predisposing causes. The intimate structure of the teeth has also an influence; predominance of mineral element giving hard, firm, dense, compact teeth little liable to decay; a comparative deficiency of this element gives a softer structure more liable to decay.

The author classifies the predisposing causes of this latter condition as follows:

1st—"Hereditary condition of the system, in which all the tissues of the body, are more or less imperfectly formed."

2nd—"Although the general condition of the system may be apparently good, the structure of the teeth may be imperfect, as a consequence of some hereditary defect."

3rd—"They may be defective in structure in consequence of some disordered condition of the system at the time of their formation.

The author's remarks here, are plain, simple and pointed, leading the reader to the conclusion that a critical inspection of the teeth and an accurate examination of all collateral facts as to the history of the patient are very important, and go far towards determining the prognosis and modifying the treatment of the case.

Frequent and rigid examinations of the teeth are absolutely necessary, to justly appreciate their condition and to treat them in a scientific manner. The acid found in the mouth, and by which the teeth are supposed to be corroded, may not always proceed from the mucous secretions. Amylaceous or starchy materials, as food, are transformed by the action of the saliva (at temperature 100°), 1st dextrine and glucose, 2d lactic acid resulting.

Animal matter retained between the teeth, may under the influence of the temperature and secretions of the mouth, undergo a species of chemical decomposition, giving an acid, possibly lactic, as one product.

The frequency and rapidity of decay where teeth approximate, is therefore easily explained, and the means of prevention suggested.

The secretions of the mouth, especially if rendered abnormal by any vice of the system; the different kinds of food, by decomposition of which an acid may be produced, are therefore directly or indirectly causes, predisposing, of caries. Dr. C. A. Harris, in his work quoted from above, emphatically denies that caries ever commences first in the internal structure of a tooth, unless there exist a fracture in the enamel by which the secretions of the mouth can have access to the dentine. Upon page 248 he states, "it (caries) usually commences or occurs on the outer surface of the dentine of the crown under the enamel." And explains it upon page 260 as follows: "And in the proximal sides of the teeth where the outer covering is so fractured by pressure of the organs against each other, that the juices of the mouth find ready access to the subjacent dental tissue."

We do not fully endorse either statement, and consider the fracturing at least, to be but exceptionally present. If decay occur under the enamel at all first, it is from the porous, defective nature of the enamel and not from fracturing. The enamel is more slowly, the dentine more rapidly destroyed by the agents, hence defective enamel may give only very minute carious canals as means of access to the dentine, but the dentine once reached by the fluids of the mouth, the decay spreads more rapidly and assumes a more definite character. Hence, a very minute carious opening precedes in the enamel, extensive lesion in the dentine. The one point here, is this, "decay always commences upon the exterior."

Dr. Harris speaking of artificial teeth decaying, (i. e. natural teeth, bone or ivory,) as do the natural undetached teeth, says, "and the decayed part in one, exhibits about the same characteristics as in the other." —(Page 259.)

We commend this statement to the notice and calm thought of the profession, as if it should be true absolutely, it is the death-blow of the vital theory of decay and equally so of the theory of circulation of blood itself in dentine. We are not fully prepared to give a decided opinion here, but we accept this as being comparatively true, not absolutely so. These are points for us to determine.

1st.—Artificial decay—is the process like the decay of the natural teeth?

2nd.—Does decay invariably begin upon the exterior?

3rd.—Does decay ever return in an accurately filled cavity?

Dr. L. S. Beale in his Structure, Life and Growth, pages—194—5 gives us the following:

> "A dead tooth may remain for years, perhaps firmly fixed in the socket, and it has recently been proved by the highly interesting researches of Dr. Mitscherlich that teeth which have been removed from the body, even for years, may be fixed in the alveolus of a living person, and be retained there for a long period. In this case the tooth is not renourished, but remains as lifeless as it was before. It appears that by the agency of some cells of the periodontal membrane remaining in the socket, little cavities are formed upon the surface of the old cementum and dentine, and that afterwards new cementum is produced as a counterpart, and thus the dead tooth is firmly held in its place."

Dr. Mitscherlich's paper, which contains many things of physiological interest, has been translated and published in Truman's Archives of Dentistry, No. II.

This is a strong statement and we confess to an urgent desire *to see these teeth* thus placed and forming adhesions. We would be glad to have the phenomena of decay in these teeth accurately noted and compared with the *live* teeth decaying—the phenomena in each case accurately and scientifically noted; a sort of differential diagnosis made, if there be any points of difference upon which to found such a diagnosis. Will some of the profession please try the experiment and mark the results. For by this means a great deal of light could be thrown upon the subject.

Chapter III.—"Treatment of Decay of Teeth."

Here our author following logically his theory of decay, urges the necessity of removing as far as practicable every irregularity, depression, fissure, &c., that the fluids of the mouth and particles of food may find no place of deposit; a smooth polished surface is the great desideratum, and if this can be obtained without fillings, it is well; but if not, then filling must be resorted to, and both fillings and surrounding edges of cavity finished off with the highest possible polish.

Attention to the first permanent molars is enjoined.

"The teeth which soonest decay from defects of enamel, are the first permanent molars."—(Page 19.)

"There is scarcely a child of the present day who does not lose these teeth unless they are plugged soon after they come."—(Page 20.)

This is also a strong statement, and of course the more important as it refers to the early years of life, when the parents and professional adviser are strictly accountable for the proper attention to the teeth. The extraction of these teeth is not advised, except in a very few instances; when the teeth are crowded preference should be given the molar and one of the bicuspids extracted "as these teeth are of less importance, and are more commonly lost, even after the extraction of the first molars, than any others."

"The idea of placing a child six or seven years of age, in the hands of a dentist, seems to shock the sensibilities of many persons; but it should always be remembered that the true method of avoiding painful operations on the teeth, is to give them attention as soon as the slightest evidence of decay is detected."—(Page 22.)

The question is one of interest and we unhesitatingly advocate the author's view; early attention may cause pain and suffering to a mere child, but the advantage is one too great to be casually passed over, for delay but increases the certainty of pain, its degree and intensity, while the teeth are jeopardized.

(To be Continued.)

---

